# The inference of HIV-1 transmission direction between HIV-1 positive couples based on the sequences of HIV-1 quasi-species

**DOI:** 10.1186/s12879-019-4163-4

**Published:** 2019-06-28

**Authors:** Jianjun Wu, Zhongwang Hu, Hui Yao, Hai Wang, Yanhua Lei, Ping Zhong, Yi Feng, Hui Xing, Yuelan Shen, Lin Jin, Aiwen Liu, Yizu Qin, Lifeng Miao, Bin Su, Yibo Zhang, Hongxiong Guo

**Affiliations:** 1 Anhui Provincial Center for Disease Control and Prevention, 12560 Fanhuadadao, Hefei, China; 2 Hefei Prefecture Center for Disease Control and Prevention, 86 Liu’an Road, Hefei, China; 3 Shanghai Municipal Center for Disease Control and Prevention, 1380 Zhongshan West Road, Shanghai, China; 4 Chinese Center for Disease Control and Prevention, 155 Changbei Road, Beijing, China; 5 Department of Hospital Infection Control, Shanghai Ruijin Hospital Affiliated to Shanghai Jiao Tong University School of Medicine, Shanghai, 200025 China; 6 Jiangsu Provincial Center for Disease Control and Prevention, 172 Jiangsu Road, Nanjing, China

**Keywords:** HIV, Transmission direction, Quasi-species, Phylogenetic analysis, Source identification of HIV infection

## Abstract

**Background:**

To infer transmission direction of a HIV transmission chain is helpful not only in legal jurisdiction but also in precise intervention to prevent HIV spread. Recently, the direction of transmission is inferred by whether paraphyletic-monophyletic (PM) or a combination of paraphyletic and polyphyletic (PP) topologies is observed or not between the sequences of source and recipient in the phylogenetic tree. However, paraphyly between them often declines over time and may disappear between spouses due to bidirectional transmission after primary infection. In this study, our aim is to test the reliability of inferring HIV transmission direction between epidemiologically linked HIV-1 positive couples using whether or not paraphyly is observed in phylogenetic tree.

**Methods:**

HIV quasi-species were sequenced using PCR product clones, and then Bayesian analysis of molecular sequences with MCMC was employed to construct phylogenetic relationship of *env, gag, pol* gene fragments of HIV-1 positive couples using BEAST software.

**Results:**

Our results showed that all sequences of seven couples except *pol* sequences of couple 12 and 13 form their own monophyletic cluster in phylogenetic tree including the closest control sequences from GenBank or other studies on local samples, which are supported by significant Bayesian posterior probabilities more than 0.9932. Of seven couples, paraphyly is only observed in phylogenetic tree constructed with *env* and *po*l gene sequences of three couples and *gag* gene sequences of four couples. Paraphyly is not observed in half of HIV positive couples. *Pol* sequences of couple 13 is separated by Blast selected controls; *pol* sequences of couple 12 in phylogenetic tree is supported by a lower Bayesian posterior value.

**Conclusion:**

Paraphyly relationship between sequences of donator and recipient is only observed among partial HIV-1 positive couples with epidemiological link. Phylogenetic relationship is not always the same when various gene regions of HIV are used to conduct phylogenetic analysis. The combination of phylogenetic analysis based on various gene regions of HIV and enough epidemiology investigation is essential when inferring transmission direction of HIV in a transmission chain or in one couple. However, while observed paraphyly can be used to infer transmission direction in HIV-1 positive couple, no observed paraphyly cannot deny it.

## Background

Phylogenetic inference of microorganisms’ transmission routes helps humans to understand epidemiologic dynamics of specific microorganism between various regions and species [1–4]. For those viruses with the ability of high diversity, phylogenetic reconstruction is often used to interpret transmission events. For example, phylogenetic analysis was used to not only identify the origin of high pathogenic influenza virus when high pathogenic influenza virus is present, but also to interpret HIV transmission route between various regions or provinces in a country [5, 6]. HIV transmission among various individuals is often involved in legal disputes between donator and recipient [7, 8]. In some cases, phylogenetic relation of HIV sequences from doubtful donator and recipient was central to the evidence of guilt [9]. Especially, to infer the transmission direction in a transmission chain is vital in these legal cases. Moreover, the interpretation of phylogenetic trees has a broader importance beyond criminal investigations, especially in public health investigations and practices.

Between two epidemiologically linked HIV-1 positive individuals, three possible ways of transmission exist. The first is direct transmission, the second is that there is one intermediary between two HIV-1 positive individuals, the last is that two HIV-1 positive individuals share a common infection source. To infer transmission direction between them, Yang et al. consecutively sampled in various time points for transmission pairs and identified transmission direction by observing the coreceptor switch of HIV from CCR5 to CXCR4 in vivo. Although the accuracy of this method, is up to 94.5%, it takes a long time to identify the direction between transmission pairs, and is only applicable to those viruses using CCR5 coreceptor in the early phase of infection [10]. Therefore, the more common method is to reconstruct phylogenetic relationship using the samples collected at the same time point.

In phylogenetic tree, the paraphyly relationship exists between the donor’s sequences and the recipient’s [11, 12]. Consequently, the transmission direction was inferred [13]. However, the paraphyly relationship between donor’s sequences and the recipient’s often decreases over time because evolution of HIV-1 in vivo in different bodies [11]. Moreover, the evolution of the various gene regions of HIV faces different pressure and experiences different models in vivo [13]. Which implies paraphyly relationship is not always observed, and paraphyly relationship is inconsistent between various gene regions of among epidemiologically linked HIV-1 positive pairs. In this study, we selected seven HIV-1 positive couples to explore the phylogenetic relationship of HIV among them and to analyze the feasibility of using observed paraphyly to infer transmission direction.

## Methods

### Epidemiological data and samples

#### Design

Firstly, to select HIV positive spouses, where only one party of HIV positive spouse has HIV risk behavior including sexual transmission and drug using, and the other party is infected with HIV through sexual transmission with her/his spouse. Secondly, to amplify the fragment of *env, gag, pol* gene, and to construct the clone of PCR amplificons. The various bacteria clone transinfected with PCR amplificons were sequenced to analyze HIV quasi-species in spouse. Finally, the paraphyletic relationship between HIV quasi-species sequences from spouse in MCMC phylogenetic tree was observed.

#### Subjects

Seven HIV-1 positive couples were investigated for their sexual behaviors which includes probable infectious route, the history of extramarital sexual behaviors, and history of intravenous drug injection. None knew their own and corresponding spouse’s status of HIV infection before confirmed HIV infection.

#### Samples

Whole blood samples were collected using sterile ethylenediaminetetra-acetic acid tubes. The plasma was centrifugally separated at 3000 rpm within 6 h, then kept at − 80°Cfor viral RNA extraction. The study was reviewed and approved by the ethical committee at the Anhui Center for Disease Control and Prevention. Written informed consent was obtained from all participants after we informed them of the objective of this study.

### RNA extraction, PCR, Clone,and sequencing

Viral RNA was extracted from 140 μL of plasma using QIAamp Viral RNA Mini kit (Qiagen, Valencia, CA). HIV-1 segments of *env*(C2-V3), *gag* (p17 and partial p24) and *pol* (protease and p51RT) were amplified using reverse transcriptase (RT)-nested polymerase chain reaction (PCR). The first PCR reactions were performed using the Superscript TM III one-step RT-PCR system with platinum Taq DNA polymerase (Invitrogen) with outer primer pair gp41-1 s/gp41-2as-B, GAG-L/GAG-E2, and MAW26/RT21 to amplify *env, gag, pol* region of HIV-1, respectively.

The second PCR reactions were performed using the TaKaRa ExTaq kit (TaKaRa Biotechnology Co. Ltd., Dalian, China) with inner primer pair gp41-3 s/gp41-4as-xw, GAGF2/c-gag, and PRO-1/RT20 to amplify *env, gag,* and *pol* region of HIV-1, respectively. The sequences of primers used in this study have been previously described in detail [14, 15]. Amplified PCR products were separated on an agarose gel and purified with QIAquick gel extraction kit (Qiagen, Valencia, CA). Purified products were cloned into pTV 118 N DNA plasmid, and transfected into *E. Coli* and cultured overnight, then selected 10 bacteria colonies to sequence DNA directly using an automated ABI 3730/3730xl DNA analyzer by Beijing Biomed BioTechnologies Co., Ltd.

### Phylogenetic analysis

The clone sequences of every couple were used to search GenBank and aligned with the local sequences in other studies using BLAST program to identify the best matching HIV-1 RNA sequences. BLAST score significance was the criterion for selection. We rationalized that finding the highest matching HIV-1 sequences would increase the chances of refuting the priori hypothesis that couple’s sequences form a monophyletic clade. The reference sequences included the best matching HIV-1 RNA sequences using BLAST program and the local control sequences. The local control sequences include all sequences that were gotten in previous studies, molecular epidemiology investigation and drug resistance surveillance. Nucleotide sequences were aligned with reference strains using the Clustal X2, and phylogenetic analyses were performed in BEAST v.1.82. To assess the appropriate model of evolution for the phylogenetic analysis of the *env, gag, pol* gene datasets, likelihood ratio tests were conducted using jModelTest software. Both the general time-reversal (GTR) and Hasegawa-Kishino-Yano nucleotide substitution models with a gamma distribution model of among site rate heterogeneity were employed. The Markov chain Monte Carlo (MCMC) search was run for 5×10^6^ generations with trees sampled every 100th generation. Burn-in was set at 20% and a posterior consensus tree generated from 50,000 trees sampled. The MCMC output was tested for convergence and effective sample size using Tracer v1.4.

## Result

### Subjects’ basic characteristics

Of all men among seven HIV-1 positive couples, five males of them had the experience of extramarital sexual behavior including commercial sex activity; one was an intravenous drug user. Only one female had extramarital sexual behavior and is a Dai nationality (shown in Table 1). Among them, only one side of every couple had the risk behavior causing HIV infection before marriage. Six of these couples, husband and wife were confirmed HIV infection within 1 month, and only one within 8 months.

**Table 1:** The basic information of seven couples

Code	Sex	Diagnosis date	The history of extramarital sex behavior	Ethnic group
3	Female	Feb 22,2011	No	Han
	male	Jan 27,2011	Yes	Han
7	Female	Mar 20,2013	Yes	Dai
	male	Mar 20,2013	Yes	Han
8	Female	Sep 18,2013	No	Han
	male	Sep 23,2013	Yes	Han
9	Female	Feb 28,2013	No	Han
	male	Jan 22,2013	Yes	Han
10	Female	Nov 15,2013	No	Han
	male	Oct 24,2013	Yes	Han
12	Female	Jan 2,2104	No	Han
	male	Sep 1,2014	Intravenous drug user	Han
13	Female	Dec31,2014	No	Han
	male	Dec31,2014	Yes	Han

### Phylogenetic relationship of various gene region of HIV-1 positive couples

**Fig. 1:**
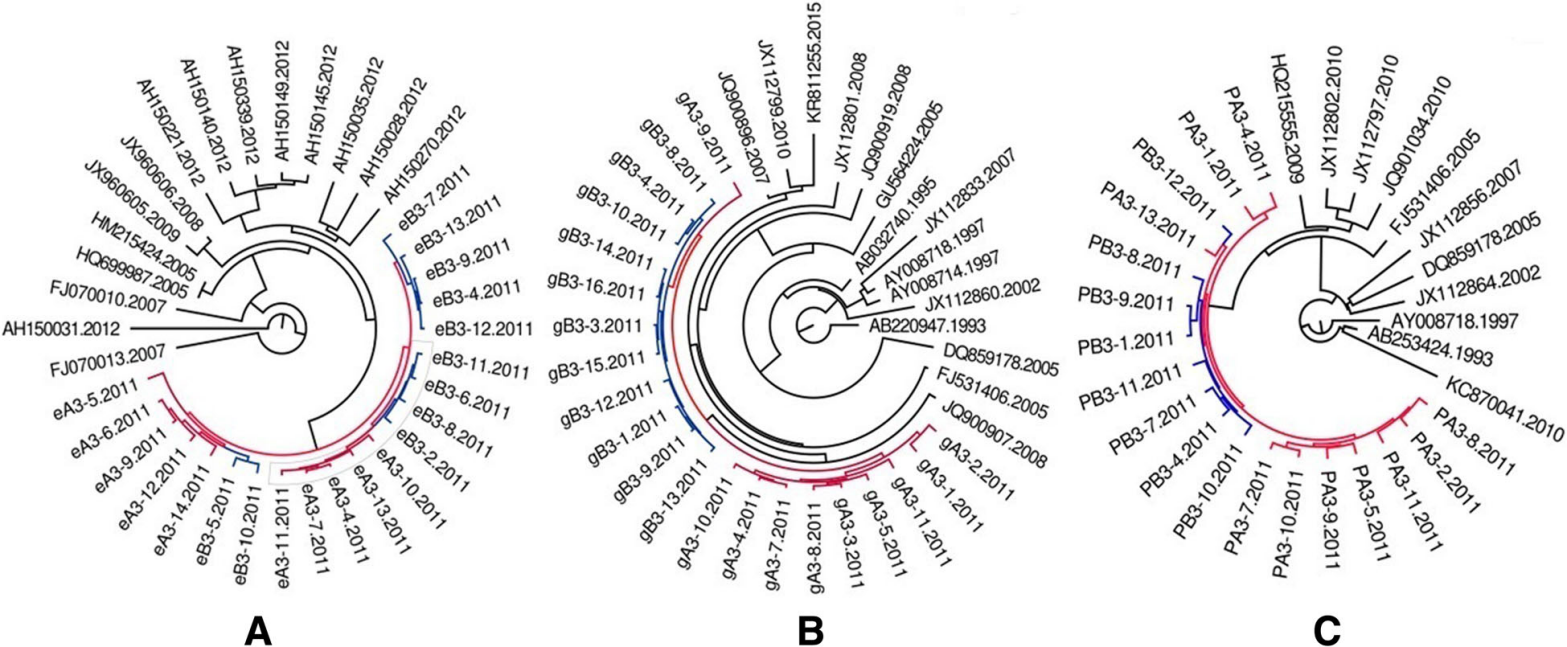
MCMC tree for the env, gag, and pol gene dataset of couple 3 using BLAST-selected GenBank and local controls in A, B, and C, respectively. The red indicates the sequences of husband while the blue is his wife’s. The black represents control sequences

**Fig. 2:**
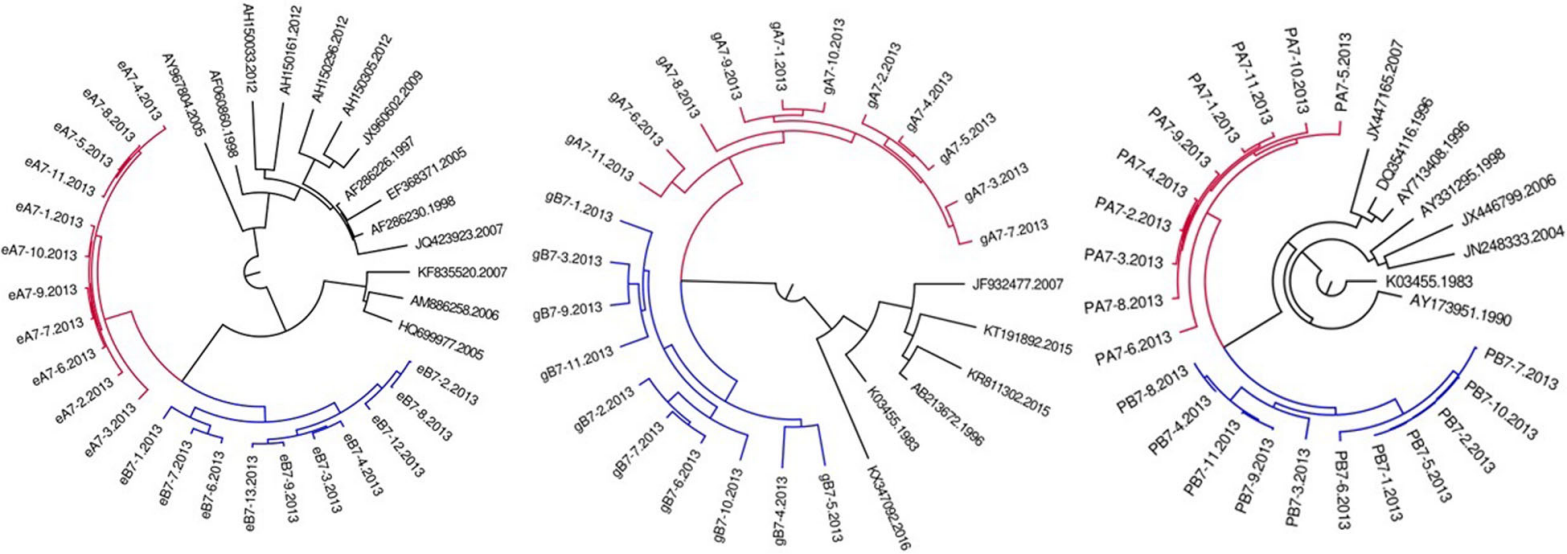
MCMC tree for the env, gag, and pol gene dataset of couple 7 using BLAST-selected GenBank and local controls in A, B, and C, respectively. The red indicates the sequences of husband while the blue is his wife’s. The black represents control sequences

**Fig. 3:**
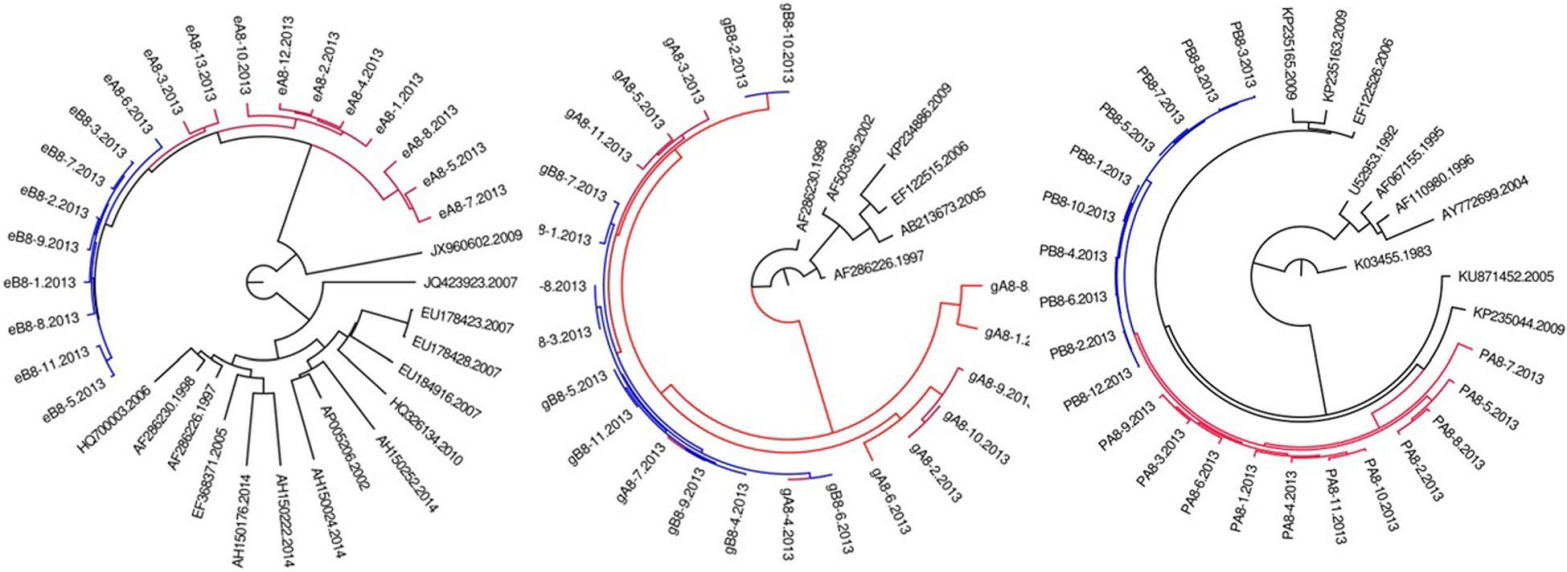
MCMC tree for the env, gag, and pol gene dataset of couple 8 using BLAST-selected GenBank and local controls in A, B, and C, respectively. The red indicates the sequences of husband while the blue is his wife’s. The black represents control sequences

**Fig. 4:**
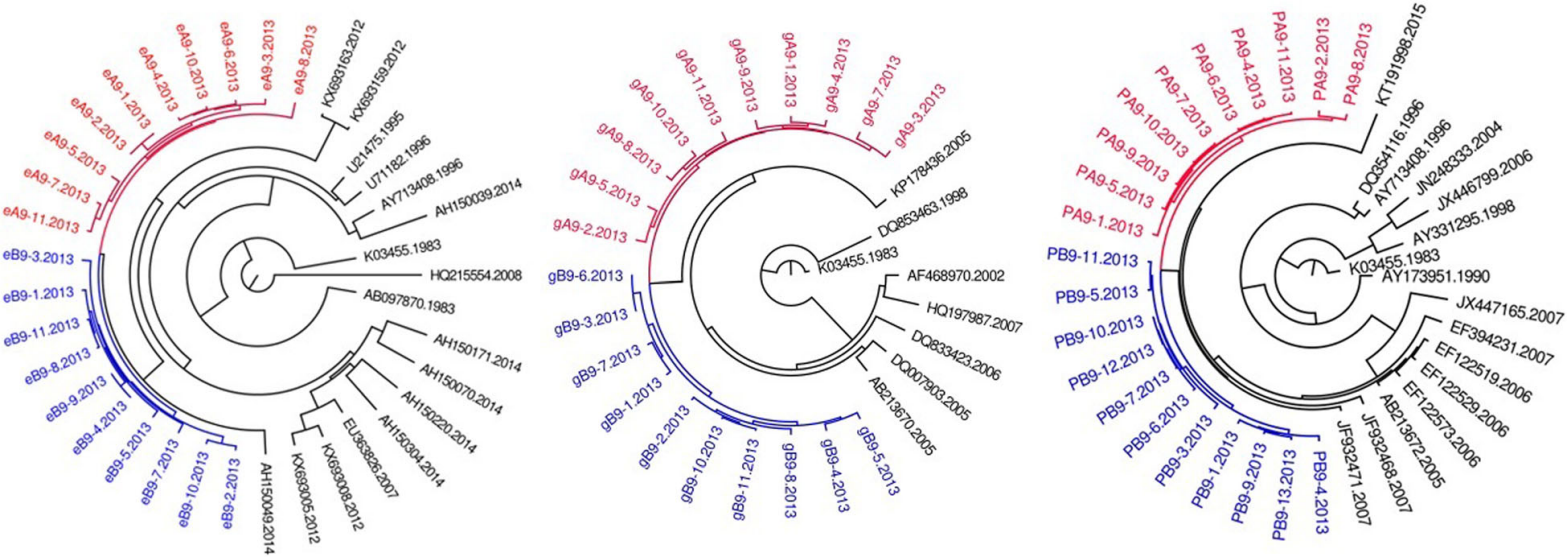
MCMC tree for the env, gag, and pol gene dataset of couple 9 using BLAST-selected GenBank and local controls in A, B, and C, respectively. The red indicates the sequences of husband while the blue is his wife’s. The black represents control sequences

**Fig. 5:**
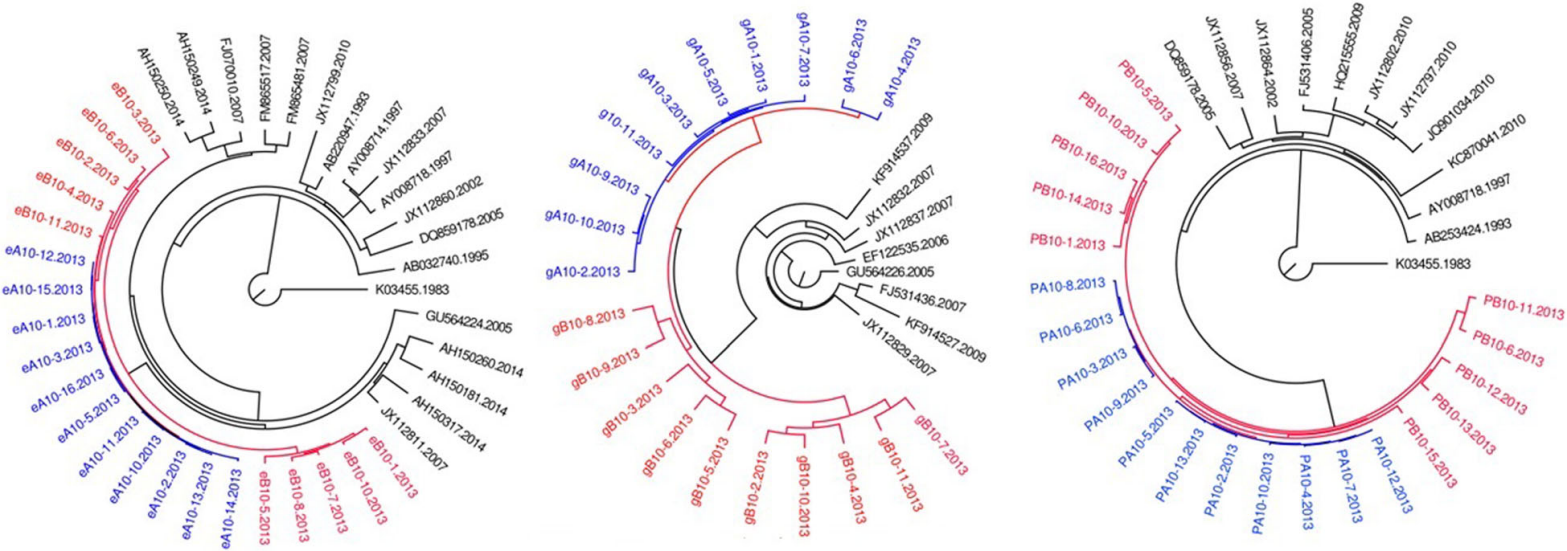
MCMC tree for the env, gag, and pol gene dataset of couple 10 using BLAST-selected GenBank and local controls in A, B, and C, respectively. The red indicates the sequences of husband while the blue is his wife’s. The black represents control sequences

**Fig. 6:**
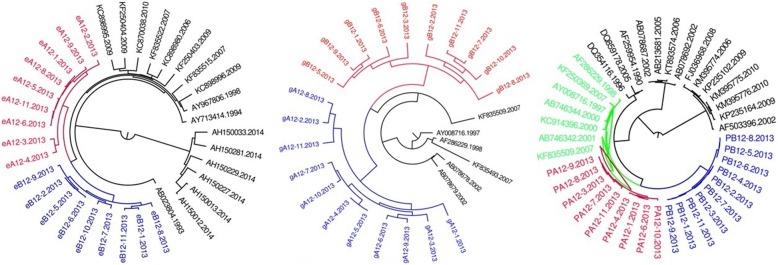
MCMC tree for the env, gag, and pol gene dataset of couple 12 using BLAST-selected GenBank and local controls in A, B, and C, respectively. The red indicates the sequences of husband while the blue is his wife’s. The black and emerald represents control sequences

**Fig. 7:**
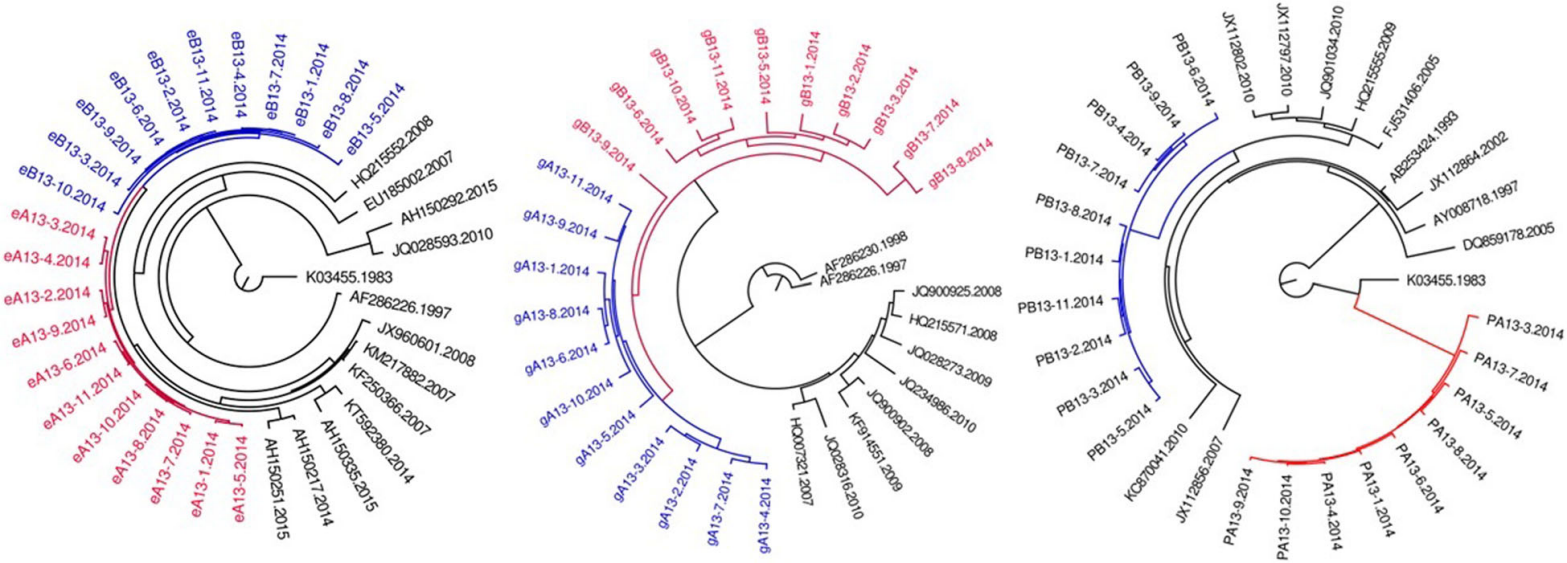
MCMC tree for the env, gag, and pol gene dataset of couple 13 using BLAST-selected GenBank and local controls in A, B, and C, respectively. The red indicates the sequences of husband while the blue is his wife’s. The black represents control sequences

All HIV-1 sequences of couple 3,7,8,9, and 10 clusters a monophyly clade with a well support of Bayesian posterior probability (as showed in Figs. 1, 2, 3, 4, and 5), respectively. As of couple 12 and 13, the sequences of *env* and *gag* formed a monophyly clade (Figs. 6a, b, 7a, and b) while the sequences of *pol* of couple 12 and 13 are separated by blast selected controls (Figs. 6c, and 7c).

As summarized in Table 2, among six of seven HIV-1 positive couples, the phylogenetic relationship between HIV sequences of couple is in accordance with in *env, gag pol*. Only couple 13, the phylogenetic relationship in *gag* region is not accordance with that of *env* and *pol*. Of them, three are PM, three are MM, one is PM in gag while MM in env and pol.

**Table 2:** The phylogenetic relationship between HIV-1 positive couples

Code	Gene Region					
	env		gag		pol	
	Phylogenetic relationship	Bayesian posterior probability	Phylogenetic relationship	Bayesian posterior probability	Phylogenetic relationship	Bayesian posterior probability
3	PM	0.9998	PM	0.9986	PM	0.9999
7	MM	0.9998	MM	0.9932	MM	0.9932
8	PM	0.9997	PM	0.9998	PM	0.9978
9	MM	0.9997	MM	0.9997	MM	0.9971
10	PM	0.9997	PM	0.999	PM	0.9981
12	MM	0.9997	MM	0.9941	MM	0.6431
13	MM	0.9997	PM	0.9997	MM	–

## Discussion

In this study, seven epidemiologically linked HIV-1 positive couples were used to infer phylogenetic relation based on quasi-species sequences of HIV-1. Epidemiology investigation confirms that only one side of every couple experienced high-risk behavior associated with HIV-1 infection. The other side of every couple has the unique chance to get HIV infection from his/her spouse. Phylogenetic analysis also shows that all sequences of *env* and *gag* region of HIV-1 form a monophyly with respect to controls, which supports that HIV-1 infected each couple shares with the most recent common ancestor and is accordance with the results of epidemiological investigation.

In previous studies, source in a transmission chain was inferred based on observed paraphyly between source and recipient with respect to controls [13]. In our study, paraphyly between source and recipient was only observed in three of seven couples amongst *env, gag* and *pol* phylogenetic trees. In couple 13, paraphyly was only observed in *gag* phylogenetic tree. The rest of them are monophyletic, using the sequences of *env, gag* or *pol* of HIV-1. Our findings indicate that paraphyly is not always observed between source and recipient. As we knew, the survival of HIV-1 in vivo experienced pressures from many aspects such as host’s immune system, antiretroviral therapy, which will lead to the loss of HIV diversity in vivo [16–21]. Therefore, paraphyly of source sequences with respect to recipient sequences will decline over time. It suggested that source can be inferred based on the observed paraphyly, and not be denied when not observing paraphyly. Our finding is consistent with the results by Diane et al [13]. In addition, recombination among viral sequences within the source individual will degrade support for particular paraphyletic relationships over time. In this study, we did not observe paraphyly between source and recipient among less than half of HIV-1 positive couples. As of HIV-1 positive couple living together for long term, HIV-1 transmission should be directional. In this occasion, it become more difficult to infer transmission direction using phylogenetic method. Moreover, it implies that half of persons were untested during the window periods of acute infection.

For most of HIV-1 couples, the phylogenetic relationship on *env, gag, pol* is consistent (couple 3, 8, 9), and with a support of higher priority value. However, there is always an exception. For example, although the *pol* sequences of couple 12 form a monophyly with respect to controls, the value of Bayesian posterior probability is very low (only 0.6431). Moreover, paraphyly is observed in phylogenetic tree based on *gag* region sequences of couple 13 while not appearing in that based on *env* and *pol*. Furthermore, the *pol* sequences of couple 13 are separated by Blast selected controls. The selection pressures that various gene region of HIV face in vivo are different. As we knew, the selective pressure which HIV faces in vivo is different between various gene regions. The most selective pressure was observed in *env* region, followed by *pol* region. *Gag* region is relatively conservative. Therefore, it is necessary to conduct phylogenetic analysis on various gene regions of HIV at the same time during inferring transmission direction.

## Conclusions

Paraphyly relationship between sequences of donator and recipient is a vital indication to infer transmission direction in HIV transmission chain. However, it is not applicable to all HIV transmission chains due to loss of paraphyly after infection for some time, especially for those spouse or sex partners living together, and intravenous drug users sharing common syringe over a long term [22]. Moreover, phylogenetic relationship is not always same when various gene regions of HIV are used to conduct phylogenetic analysis. Therefore, the combination of phylogenetic analysis based on various gene regions of HIV and enough epidemiology investigation is essential when inferring transmission direction of HIV in a transmission chain or only a couple.

## Data Availability

The datasets used in this study are available from the corresponding author on reasonable request.

## Abbreviations

BEAST: Bayesian evolutionary analysis sampling tree
GTR: General time-reversal
HCV: Hepatitis C virus
HIV: Human immunodeficiency virus
HIV-1: Human immunodeficiency virus type one
MCMC: Markov chain Monte Carlo
MEGA: Molecular evolutionary genetics analysis
